# Ancestry‐informed fine‐mapping and sequence analysis of the *BIN1* locus pinpoints rs6733839 as a causative variant shared between individuals of European and African ancestry with non‐Mendelian early‐onset Alzheimer's Disease

**DOI:** 10.1002/alz70855_107351

**Published:** 2025-12-24

**Authors:** Nicholas R. Ray, Jiji T. Kurup, Joseph Bradley, Gary W Beecham, Carlos Cruchaga, Christiane Reitz

**Affiliations:** ^1^ Columbia University Irving Medical Center, New York, NY, USA; ^2^ Washington University School of Medicine, St. Louis, MO, USA; ^3^ Wake Forest University School of Medicine, Winston‐Salem, NC, USA

## Abstract

**Background:**

Genome‐wide association studies (GWAS) of complex traits often implicate genetic loci spanning thousands of variants. Statistical fine‐mapping refines a GWAS locus to a smaller set of likely causal variants facilitating interpretation and functional validation. *Ancestry‐informed* fine‐mapping can further improve resolution by capitalizing on the genomic diversity across ancestries (e.g., smaller LD blocks in African populations). We employed cross‐ancestry fine‐mapping at top loci recently identified in a transethnic meta‐analysis of non‐Mendelian early‐onset Alzheimer's Disease (EOAD; Bradley et al. 2025) to identify population‐specific causative variants associated with this type of AD.

**Method:**

Bradley et al. (2025) identified 13 distinct disease‐associated loci, 8 of these for the first time reported in EOAD. Capitalizing on the individual ancestry‐specific summary statistics from individuals of European (NHW) and African American (AA) ancestry (NHW: 6,282 cases, 13,386 controls; AA: 782 cases, 3,663 controls) for the top loci, we employed MESuSiE to model shared and ancestry‐specific causal variants. MESuSie accounts for LD structure in multiple ancestries and accommodates causal effect size similarity and heterogeneity. Loci prioritized by MESuSiE were followed up in whole‐genome sequencing data of NHW (*N* = 6,225) and AA individuals (*N* = 4,376) from the ADSP.

**Result:**

Multi‐ancestry fine‐mapping identified regulatory variant rs6733839 in *BIN1* (transethnic EOAD GWAS *p*‐value: 1.73E‐10) as the most likely causative variant associated with EOAD at this locus and suggests that it is shared across NHW and AA populations (PIP EUR: 0.0002; PIP AFR: 0.0001; PIP EUR_AFR: 0.99). Notably, while the associated haplotypes in both ancestries overlapped, the AA haplotype was much narrower, in line with the smaller extent of LD in individuals of African ancestry and pinpointing a much more concise locus harboring the causative variant. Individual analysis of WGS data from individuals of European and African ancestry from the ADSP validated rs6733839 as disease‐associated in both ancestral population groups.

**Conclusion:**

Multi‐ancestry fine‐mapping pinpoints rs6733839 as a likely causative variant associated with non‐Mendelian EOAD and suggests that it is shared across individuals of European and African ancestry. These findings elucidate the etiology of this type of AD in these populations and demonstrate the value of ancestry‐informed fine‐mapping for identification of population‐specific causative variants.

